# Integrated Transcriptomic and Metabolomic analysis reveals a transcriptional regulation network for the biosynthesis of carotenoids and flavonoids in ‘Cara cara’ navel Orange

**DOI:** 10.1186/s12870-020-02808-3

**Published:** 2021-01-07

**Authors:** Haipeng Zhang, Jiajing Chen, Zhaoxin Peng, Meiyan Shi, Xiao Liu, Huan Wen, Youwu Jiang, Yunjiang Cheng, Juan Xu, Hongyan Zhang

**Affiliations:** Key Laboratory of Horticultural Plant Biology (Ministry of Education), College of Horticulture and Forestry, Huazhong Agricultural University, Wuhan, 430070 People’s Republic of China

**Keywords:** Citrus, Carotenoid, Flavonoid, Transcription factor, Transcriptome, Metabolomics

## Abstract

**Background:**

Carotenoids and flavonoids are important secondary metabolites in plants, which exert multiple bioactivities and benefits to human health. Although the genes that encode carotenogenesis and flavonoid biosynthetic enzymes are well characterized, the transcriptional regulatory mechanisms that are related to the pathway genes remain to be investigated. In this study, ‘Cara cara’ navel orange (CNO) fruit at four development stages were used to identify the key genes and TFs for carotenoids and flavonoids accumulation.

**Results:**

In this study, CNO was used to investigate the profiles of carotenoids and flavonoids by a combination of metabolomic and transcriptomic analyses. The important stage for the accumulation of the major carotenoid, lycopene was found to be at 120 days after florescence (DAF). The transcripts of five carotenogenesis genes were highly correlated with lycopene contents, and 16, 40, 48, 24 and 18 transcription factors (TFs) were predicted to potentially bind 1-deoxy-D-xylulose-5-phosphate synthase (*DXS1*), deoxyxylulose 5-phosphate reductoisomerase (*DXR*), geranylgeranyl diphosphate synthase (*GGPPS2*), phytoene synthase (*PSY1*) and lycopene β-cyclase (*LCYB*) promoters, respectively. Narirutin was the most abundant flavonoid in the flesh at the early stages, 60 DAF was the most important stage for the accumulation of flavonoids, and 17, 22, 14, 25, 24 and 16 TFs could potentially bind phenylalanine ammonia-lyase (*PAL-1* and *PAL-4*), 4-Coumarate-CoA ligase (*4CL-2* and *4CL-5*), chalcone synthase (*CHS-1*) and chalcone isomerase (*CHI*) promoters, respectively. Furthermore, both sets of 15 candidate TFs might regulate at least three key genes and contribute to carotenoids/flavonoids accumulation in CNO fruit. Finally, a hierarchical model for the regulatory network among the pathway genes and TFs was proposed.

**Conclusions:**

Collectively, our results suggest that *DXS1*, *DXR*, *GGPPS2*, *PSY1* and *LCYB* genes were the most important genes for carotenoids accumulation, while *PAL-1*, *PAL-4*, *4CL-2*, *4CL-5*, *CHS-1* and *CHI* for flavonoids biosynthesis. A total of 24 TFs were postulated as co-regulators in both pathways directly, which might play important roles in carotenoids and flavonoids accumulation in CNO fruit.

**Supplementary Information:**

The online version contains supplementary material available at 10.1186/s12870-020-02808-3.

## Background

Citrus is one of the most important fruits in the world, and is rich in various primary and secondary metabolites, such as amino acids, organic acids, soluble sugars, flavonoids, volatile terpenoids, limonoids, coumarins and carotenoids [1–5]. Some metabolites, including flavonoids, limonoids, organic acids and soluble sugars, were reported to contribute to fruit taste [3, 4], whilst terpenoids and carotenoids were associated with fruit aroma and color [2, 6].

Carotenoids not only contribute to the eye-attracting fruit flesh color, but also exhibit various bioactivities in human body, such as prevention of cancer and other diseases [7] and acting as the precursors for vitamin A [8]. In citrus, the flesh color mainly depends on the contents of carotenoids [6], with flavonoids and anthocyanins exerting yellow background and blood orange respectively [9, 10]. The contents of major carotenoids are varied in different citrus species, β-cryptoxanthin is the major carotenoid in loose-skin mandarin (*Citrus reticulata*), violaxanthin is the most abundant in sweet oranges (*C. sinensis*), while lower levels of carotenoids were found in most of pomelo (*C. grandis*) and citrons (*C. medica*) [11]. Moreover, some citrus germplasms are rich in special carotenoids in their specific tissues. For example, ‘Red tangerine’ peel is red, with abundant β-citraurin [12]; The flesh of ‘Hong Anliu’ sweet orange (*C. sinensis*) and ‘Cara cara’ navel orange (*C. sinensis*, CNO) is pink or red due to their high concentrations of lycopene [6]. Lycopene is an excellent antioxidant that plays important roles in skin protection, cardiovascular disease prevention, and anti-aging and anti-cancer activities [13]. A high concentration of lycopene in citrus fruit not only improves the fruit quality but is also beneficial to human health.

In higher plants, the isomeric C5 isopentenyl pyrophosphate (IPP) is synthesized in 2-C-methyl-D-erythritol-4-phosphate (MEP) pathway, and is used as the precursor of geranylgeranyl diphosphate (GGPP) by GGPP synthase gene, GGPPS. GGPP is the direct precursor for various carotenoids catalyzed by phytoene synthase (PSY), phytoene desaturase (PDS), ζ-carotene desaturase (ZDS), lycopene β-cyclase (LCYB), carotene isomerase (CRTISO), carotene hydroxylase (HYD), violaxanthin de-epoxidase (VDE) and zeaxanthin epoxidese (ZEP), etc. [8]. Previous studies have shown that 1-deoxy-D-xylulose-5-phosphate synthase (DXS) was an important rate-limiting enzyme in the MEP pathway in tomato, Arabidopsis and Kiwifruit [14, 15]. Besides, the deoxyxylulose 5-phosphate reductoisomerase (DXR) was shown to be rate-limiting enzyme in Arabidopsis, over-expression of *DXR* increased the content of carotenoids [16]. PSY is the key rate-limiting enzyme in the biosynthetic pathway of carotenoids in tomato and citrus [17, 18]. The contents of carotenoids were significantly increased when *PSY* was over-expressed in both tomato and citrus fruit [17, 18]. In tomato fruit, *DXS*, *PSY*, *PDS*, *ZDS* and *CRTISO* are up-regulated at the mature stage, resulting in lycopene’s over-accumulation [19]. The expression of *DXS* was positively correlated with the content of carotenoids in citrus [20]. ‘Hong Anliu’ sweet orange is a red flesh mutant from ‘Anliu’ sweet orange, with its fruit accumulating lycopene around 180 DAF [20]. It was found that some biosynthetic pathway genes and transcription factors (TFs) contributed to the accumulation of lycopene by combining transcriptome, proteomic and metabolomic analyses [17, 20, 21]. However, to our knowledge, the molecular basis of lycopene over-accumulation is still not clear.

Although the genes that encode carotenogenesis enzymes are well characterized, the transcriptional regulatory mechanisms that are related to the pathway genes remain to be investigated. TFs directly regulate the expression levels of their target genes, and take part in various biological processes in plants. Some TFs have been found to be associated with the accumulation of carotenoids, such as *TAGL1*, *FUL1/2* and *ERF6* [22–24]. Lycopene level was increased by over-expression of *TAGL1* or *PpPLENA* in tomato fruit [22–24], while decreased in *TAGL1* or *FUL1/2* loss of function mutant [24]. Some TFs can directly bind to the biosynthetic pathway genes’ promoters, then affect the accumulation of carotenoids. For example, *PIF1* and *SlMADS1* specially bound the *PSY* promoter and decreased its transcription and thus altered the carotenoids profile [25, 26], whereas *HY5* and *RIN* up-regulated the expression of *PSY* in tomato [27]. In addition, the *CrMYB68* decreased the expression levels of *CrBCH2* and *CrNCED5* in citrus [28], whist *CsMADS6* increased those of *LCYb1*, *PSY* and *PDS* in ‘Hong Anliu’ [17].

Cara cara navel orange (CNO) is a bud mutant with red-flesh derived from Washington navel orange, due to the over-accumulation of lycopene and the high content of β-carotene. Similar results were found with some red flesh grapefruits and sweet oranges, but with varied ratios of lycopene to β-carotene [6], indicating the different patterns of carotenoids within different genotypes. It was found that the expression levels of *DXS*, *HDS* and *HDR* in CNO were higher than those in Washington navel orange at the mature stage [29]. However, the reason for the up-regulation of these genes remains unknown.

Flavonoids, one of the most important secondary metabolites in citrus fruit, have multiple functions, such as anti-cancer, anti-virus, anti-inflammatory and anti-bacterial activities [30, 31]. Flavonoids mainly include flavanone, flavones, flavonol, dihydroflavonol and anthocyanin, and the flavanone glycosides are the major flavonoids in citrus fruit. Our previous study found that the contents of flavonoids in four sweet oranges tended to be stable or slightly decreased during 90–210 DAF, and that the lycopene accumulation may have direct or indirect effects on the biosynthesis of flavonoids [9]. The flavonoids are synthesized from phenylpropanoid pathway, mainly including *PAL*, *C4H*, *4CL*, *CHS* and *CHI* genes [32]. It was found that the expression levels of these genes were at high levels at 60 DFA, but simultaneously dropped to extremely low levels at 90–210 DAF in four pomelos, the key pathway genes might be regulated by some TFs [32]. Numerous TFs have been reported to regulate lignin and anthocyanin biosynthesis in phenylpropanoid pathway [33]. For example, *CsMYBF1* was found to bind to *CHS-1* promoter and then affect flavanone accumulation in citrus [34]; Ruby was found to bind to *CHS*, *DFR* and *ANS* promoters, then regulate anthocyanin accumulation in blood orange and Zipi pomelo [10, 35]. Furthermore, many MYB TFs were reported to directly bind and regulate *PAL* and *C4H* promoter to affect lignin accumulation in *Arabidopsis* and sweet orange [36, 37]. However, to our knowledge, the regulatory mechanisms of TFs in the biosynthesis of both flavonoids and carotenoids remain unclear in citrus fruit.

In this study, HPLC-based carotenoids and flavonoids profiling combined with RNA-seq based gene expression data is utilized to mine the important pathway genes and TFs contributing to carotenoids and flavonoids profiles in juice sacs during fruit development of CNO fruit. The results may provide new insights into the mechanism governing specific carotenoids and flavonoids accumulation in citrus fruit.

## Results

### Carotenoids and flavonoids profiles in juice sacs of CNO during fruit development

Juice sacs at six different developmental stages of CNO were determined for variation in carotenoids and flavonoids profiles using HPLC. The contents of both carotenoids and flavonoids varied greatly at different developmental stages.

**Fig. 1:**
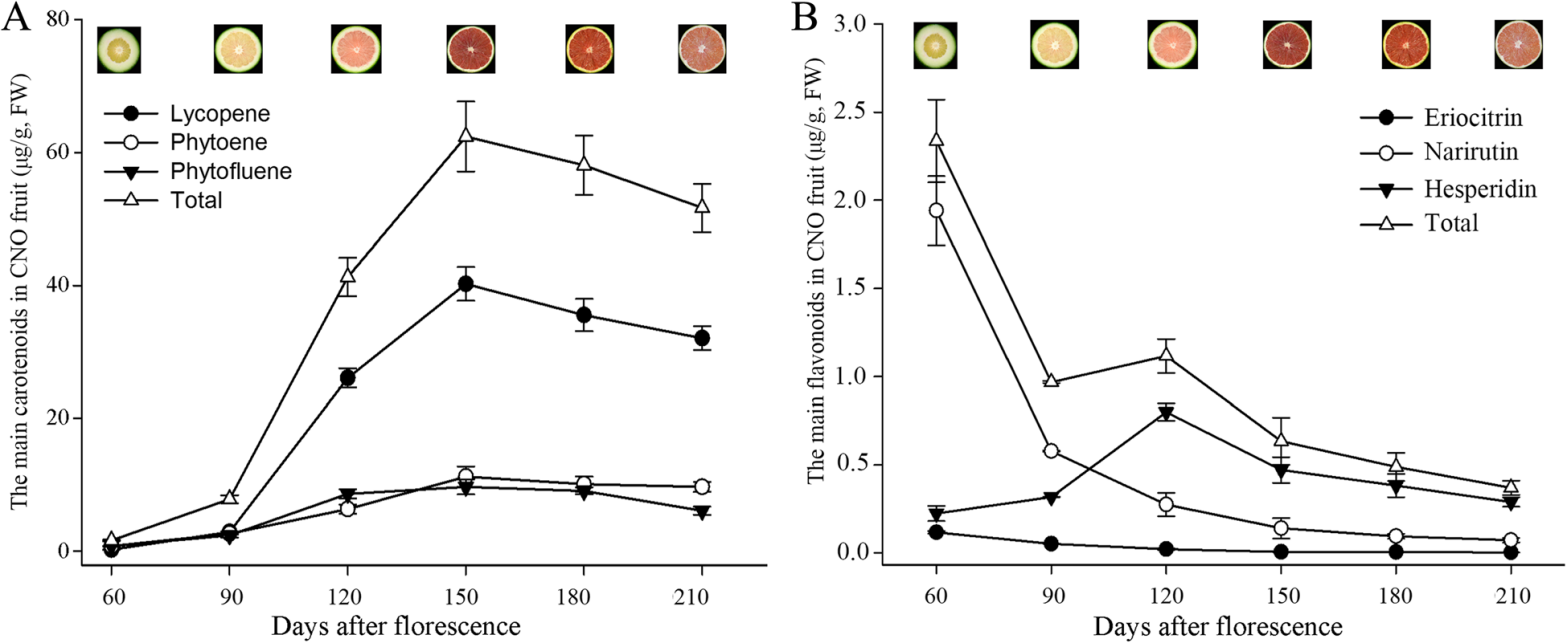
The main carotenoids and flavonoids identified in CNO juice sacs during fruit development. **a**, The main carotenoids in CNO fruit (μg/g, FW); **b**, The main flavonoids in CNO fruit (μg/g, FW)

Seven major carotenoids were identified, including lycopene, phytoene, phytofluene, lutein, violaxanthin, β-cryptoxanthin and β-carotene, with the level of lycopene being the highest (Additional file 1: Table S1; Fig. 1a). The content of lycopene was low at 60 DAF, and significantly increased from 90 to 120 DAF, reached to a maximum at 150 DAF, then significantly decreased at the mature stage (Fig. 1a). The increases of lycopene levels were 2.65 μg/g, 23.23 μg/g and 14.19 μg/g from 60 to 90, 90 to 120 and 120 to 150 DAF, respectively, indicating that the stage from 90 to 120 DAF might be the most important for the lycopene biosynthesis (Additional file 1: Table S1; Fig. 1a). For the other carotenoids identified, phytoene and phytofluene were also significantly increased from 90 to 120 DAF, then maintained or slightly decreased at the mature stage (Fig. 1a). Lutein was found at 180 DAF and its level increased at 210 DAF; β-cryptoxanthin and β-carotene were detected at 150 DAF and tended to increase from 150 to 210 DAF; violaxanthin was detected at 120 DAF, then significantly increased from 150 to 180 DAF (Additional file 1: Table S1).

A total of 11 flavonoids were identified in CNO fruit at four development stages, with narirutin as the dominant one at 60 and 90 DAF (Additional file 1: Table S2). The contents of total flavonoids were the highest and lowest at 60 DAF and 210 DAF, respectively. The results suggested that the 60 DAF stage might be the important stage for the accumulation of flavonoids (Fig. 1b).

Pearson correlation coefficient was − 0.797 between total carotenoids and total flavonoids levels in CNO fruit at six development stages, while − 0.719 was at 60 to 150 DAF, suggesting a negative correlation between total contents of carotenoids and flavonoids. Furthermore, before 150 DAF, the level of carotenoids constantly increased, whereas the content of flavonoids sharply decreased at 90 DAF with a slight increase at 120 DAF due to the increase of hesperidin content. After 150 DAF, the level of lycopene significantly decreased, whilst the levels of the other main carotenoids and all flavonoids decreased or kept at stable levels (Fig. 1). These results showed that there were collaborative changes in carotenoids and flavonoids biosynthesis, with 150 DAF as the turning stage.

### Differentially expressed genes in CNO during fruit development

**Fig. 2:**
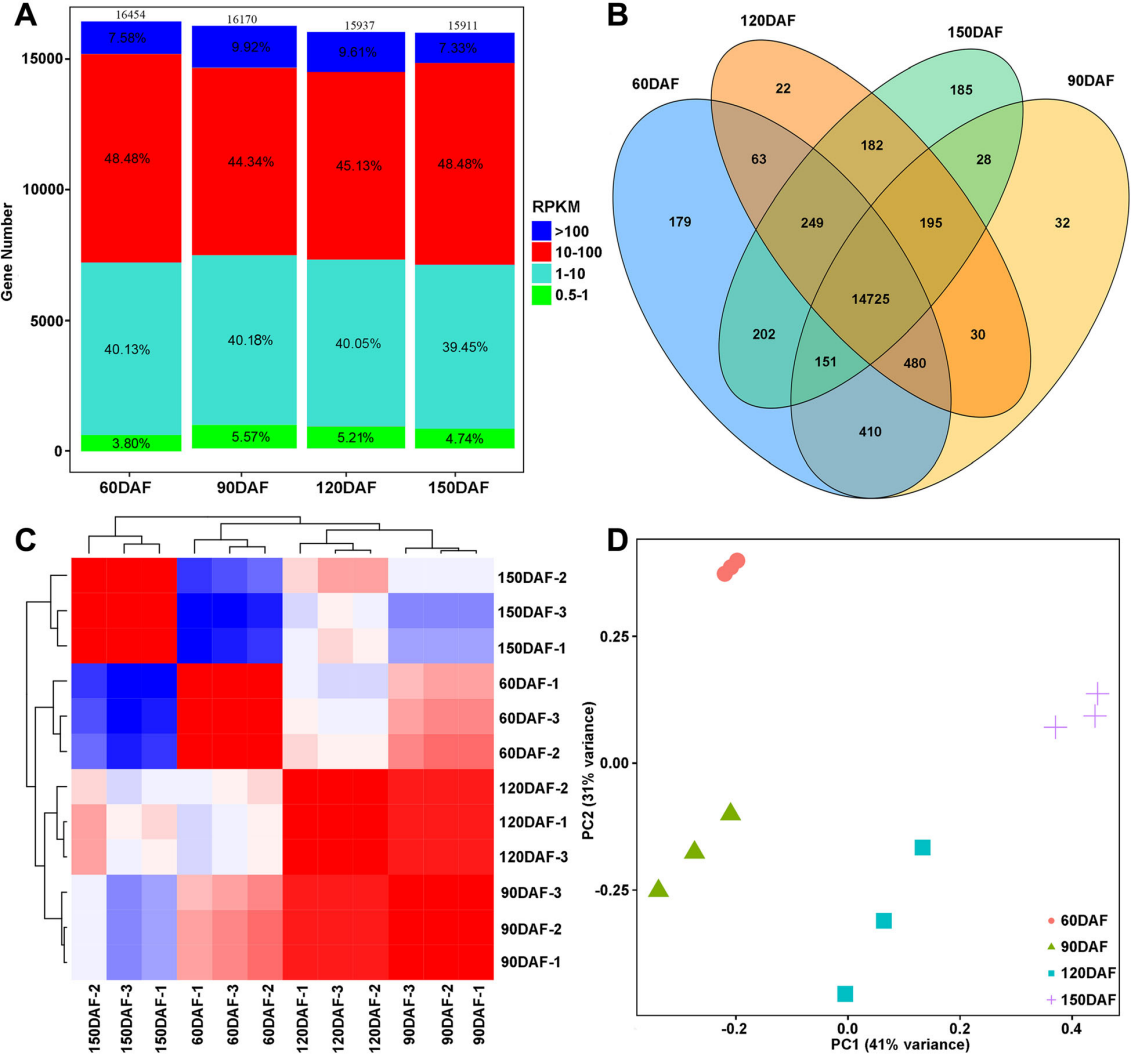
Basic information obtained from transcriptomic data. **a**, Expression levels of genes based on RNA-Seq data from juice sacs of CNO at 4 different development stages. **b**, The specially expressed genes in juice sacs of CNO at 4 different development stages. **c**: Correlation analysis matrix among CNO at 4 different development stages based on RNA-Seq data. **d**: PCA analysis on CNO at 4 different development stages based on RNA-Seq data

To further determine the expression levels of the genes related to lycopene over-accumulation in CNO, the juice sacs at four developmental stages (60, 90, 120 and 150 DAF) were subjected to transcriptomic analysis. Total of 282.14 M high-quality base pairs were obtained, with an average of 23.51 M data per sample. Approximately 94.68–97.57% reads were mapped to the Valencia sweet orange genome, among them, 87.22–93.13% was uniquely aligned, and 4.44–7.46% was aligned to multiple loci (Additional file 1: Table S3). Based on their expression levels, the average fragments per kilobase of transcript per million fragments mapped (FPKM) higher than 0.5 were selected for further analysis (Additional file 1: Table S4). Totally there were 17,190 genes expressed in juice sacs at four development stages of CNO, with 16,454, 16,170, 15,937 and 15,911 expressed at 60, 90, 120 and 150 DAF, respectively (Fig. 2a), while 178, 32, 20 and 187 genes were specially expressed at 60, 90, 120 and 150 DAF, respectively (Fig. 2b). Furthermore, 88.61, 84.62, 85.18 and 87.93% of expressed genes ranged from 1 to 100 FPKM in 60, 90, 120 and 150 DAF, respectively (Fig. 2a). Principal component analysis (PCA) and correlation analysis showed good repeatability among biological replicates (Fig. 2 c, d).

A total of 2482, 4059, 5565, 3738, 5671 and 3555 differentially expressed genes (DEGs) at 60 DAF vs 90 DAF, 60 DAF vs 120 DAF, 60 DAF vs 150 DAF, 90 DAF vs 120 DAF, 90 DAF vs 150 DAF and 120 DAF vs 150 DAF with the fold change (|log2FC| ≥ 1) and FDR ≤ 0.05 were obtained, respectively. For instance, compared with the fruit at 90 DAF, 2063/1675 genes were significantly up-regulated/down-regulated in the fruit at 120 DAF (Additional file 3: Fig. S1 A).

### Important pathway genes for lycopene accumulation

The stage from 90 to 120 DAF was the most important stage for lycopene accumulation based on metabolic data. Thus, the DEGs were subjected to KEGG enrichment analysis, and the DEGs were found to be mainly enriched in metabolic pathways, biosynthesis of secondary metabolites and plant hormone signal transduction (Additional file 3: Fig. S1 B). Among the DEGs, some genes were clustered into carotenoids biosynthetic and terpenoid backbone biosynthesis, including *DXS1* (Cs1g20530), *DXS2* (Cs9g05150), *HDS* (Cs8g16700), *HDR* (Cs8g07020) and *ZDS4* (Cs3g11060), and their expression levels in the fruit at 120 DAF were significantly higher than thoseat90 DAF. Furthermore, KEGG enrichment analysis on the DEGs at 90–120 DAF found that some genes were clustered into plant hormone signal transduction, such as Cs9g08850 (ethylene signal transduction), Cs1g17210 (jasmonate signal transduction) and Cs9g18020 (abscisic acid signal transduction), etc.: which might also play important roles in lycopene over-accumulation (Additional file 3: Fig. S1 B).

**Fig. 3:**
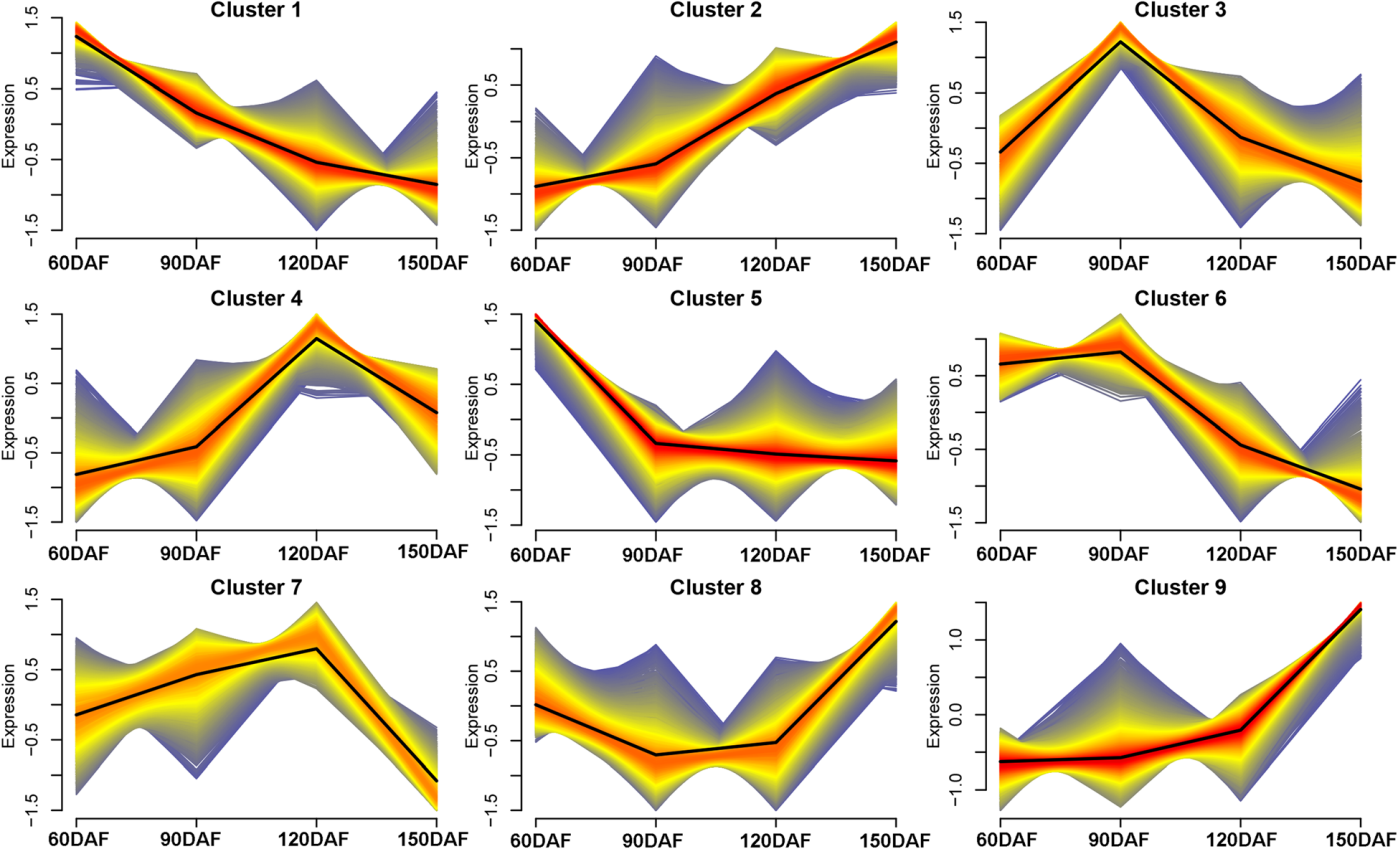
Expressed genes were clustered into nine expression patterns using Mfuzz in CNO at four developmental stages

To mine the most important genes for the over-accumulation of lycopene during fruit development, all the expressed genes were grouped using Mfuzz analysis based on their expression pattern changes, which yielded nine clusters (Fig. 3; Additional file 1: Table S5). Notably, Cluster 2, 4 and 7 showed a good positive correlation with the content of lycopene, and 1804, 1604 and 1546 genes were grouped in Cluster 2, 4 and 7, respectively (Fig. 3). Further gene function annotation found that *DXS1*, *PSY1* and *ZDS2* (Cs3g11060) were in Cluster4, *DXR* and *GGPPS2* were in Cluster2, and *MDS* (Cs5g03050) was in Cluster7, implying their importance as candidate genes accounting for lycopene over-accumulation. In addition, *LCYB* (orange1.1 t00772) with consistently low expression levels was in Cluster 6 (Fig. 3).

**Fig. 4:**
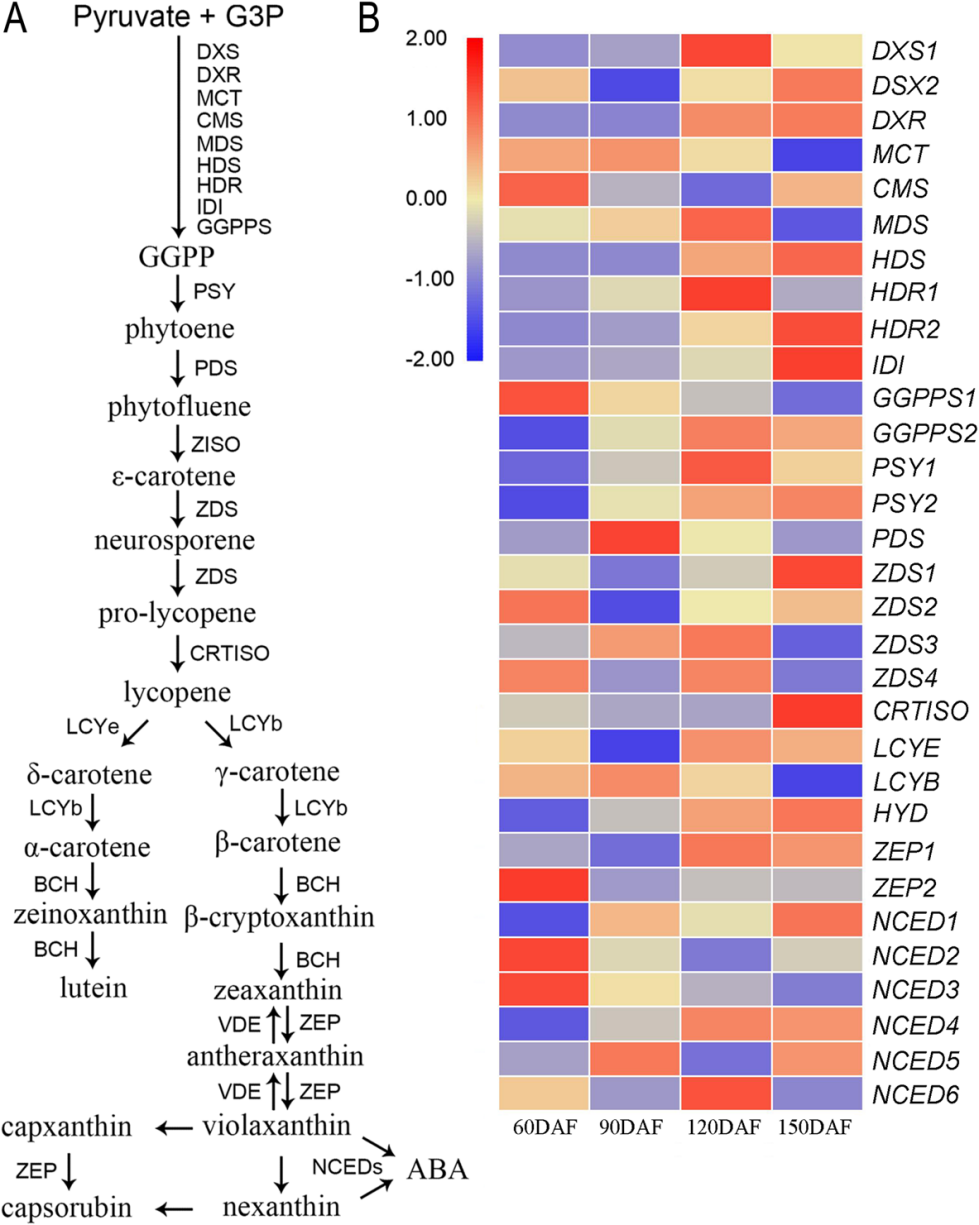
Heatmap representation of the expression levels of carotenoid biosynthesis-related genes in CNO fruit based on transcriptome data. **a**, Carotenoid metabolism pathway in citrus. **b**, Expression level of carotenoid metabolism pathway genes in CNO fruit at four developmental stages

Next, correlation analysis between the genes within carotenoid biosynthetic pathway and the content of lycopene showed that *DXS1*, *DXR*, *GGPPS2* and *PSY1* were highly and positively related to lycopene levels, with correlation coefficients of 0.997, 0.893, 0.834 and 0.964, respectively, while the values of the other lycopene biosynthesis related genes were lower than 0.800 (Table 1; Fig. 4). Herein, *DXS1*, *DXR*, *GGPPS2* and *PSY1* might be the key biosynthetic pathway genes accounting for the high level of lycopene in juice sacs of CNO fruits.

**Table 1:** Pearson correlation coefficients between the increases of lycopene levels and carotenoid biosynthetic genes

GeneID	GeneSymbol	CNO60	CNO90	CNO120	CNO150	PCC^a^
Cs1g20530	DXS1	10.89	20.19	78.62	45.66	0.997
Cs9g05150	DXS2	7.11	1.93	6.16	10.08	0.343
Cs5g05440	DXR	35.04	35.42	61.87	64.87	0.893
Cs3g01420	MCT	29.61	30.12	25.74	16.03	−0.541
Cs2g28970	CMS	40.14	26.31	21.28	33.00	−0.631
Cs5g03050	HDS	125.48	142.94	168.68	97.26	0.352
Cs8g16700	MDS	33.72	37.99	104.16	158.33	0.743
Cs5g28200	HDR1	48.45	50.28	52.16	48.77	0.793
Cs8g07020	HDR2	0.18	0.45	1.51	4.87	0.511
Cs6g02690	IDI	120.99	124.26	133.57	177.28	0.448
Cs1g23670	GGPPS1	103.41	77.77	63.99	51.92	−0.738
Cs8g02140	GGPPS2	16.90	10.97	26.47	27.88	0.834
Cs6g15910	PSY1	44.65	58.84	89.89	66.70	0.964
orange1.1 t02108	PSY2	0.53	1.34	1.53	1.70	0.784
Cs5g20250	PDS	3.45	5.41	3.99	3.42	−0.250
Cs3g11180	ZDS1	9.86	3.00	8.33	37.89	0.408
Cs3g11060	ZDS2	3.15	0.00	1.27	1.87	−0.280
orange1.1 t05048	ZDS3	2.76	3.21	6.22	1.64	−0.159
Cs3g11090	ZDS4	3.01	1.17	3.00	1.92	0.321
Cs6g13340	CRTISO	6.93	5.62	6.17	12.52	0.163
Cs4g14850	LCYE	2.48	2.66	5.38	4.94	0.943
orange1.1 t00772	LCYB	8.39	10.57	7.73	4.66	−0.476
Cs9g19270	HYD	141.38	275.22	632.9	847.80	0.805
Cs1g22620	ZEP1	5.68	3.13	23.67	19.12	0.969
orange1.1 t04050	ZEP2	4.29	1.14	1.62	1.58	−0.546
Cs2g03270	NCED1	20.98	61.70	42.32	78.31	0.322
Cs5g14370	NCED2	26.11	6.98	2.50	5.49	−0.711
Cs7g01700	NCED3	3.71	1.11	1.02	0.65	−0.744
Cs7g01710	NCED4	92.84	117.49	152.99	148.52	0.933
Cs7g14820	NCED5	6.33	13.39	5.24	11.45	−0.338
Cs8g14150	NCED6	3.52	0.89	9.75	0.67	0.661

*PCC*^*a*^: Pearson correlation coefficient

### TFs related to key carotenoid biosynthetic genes

**Fig. 5:**
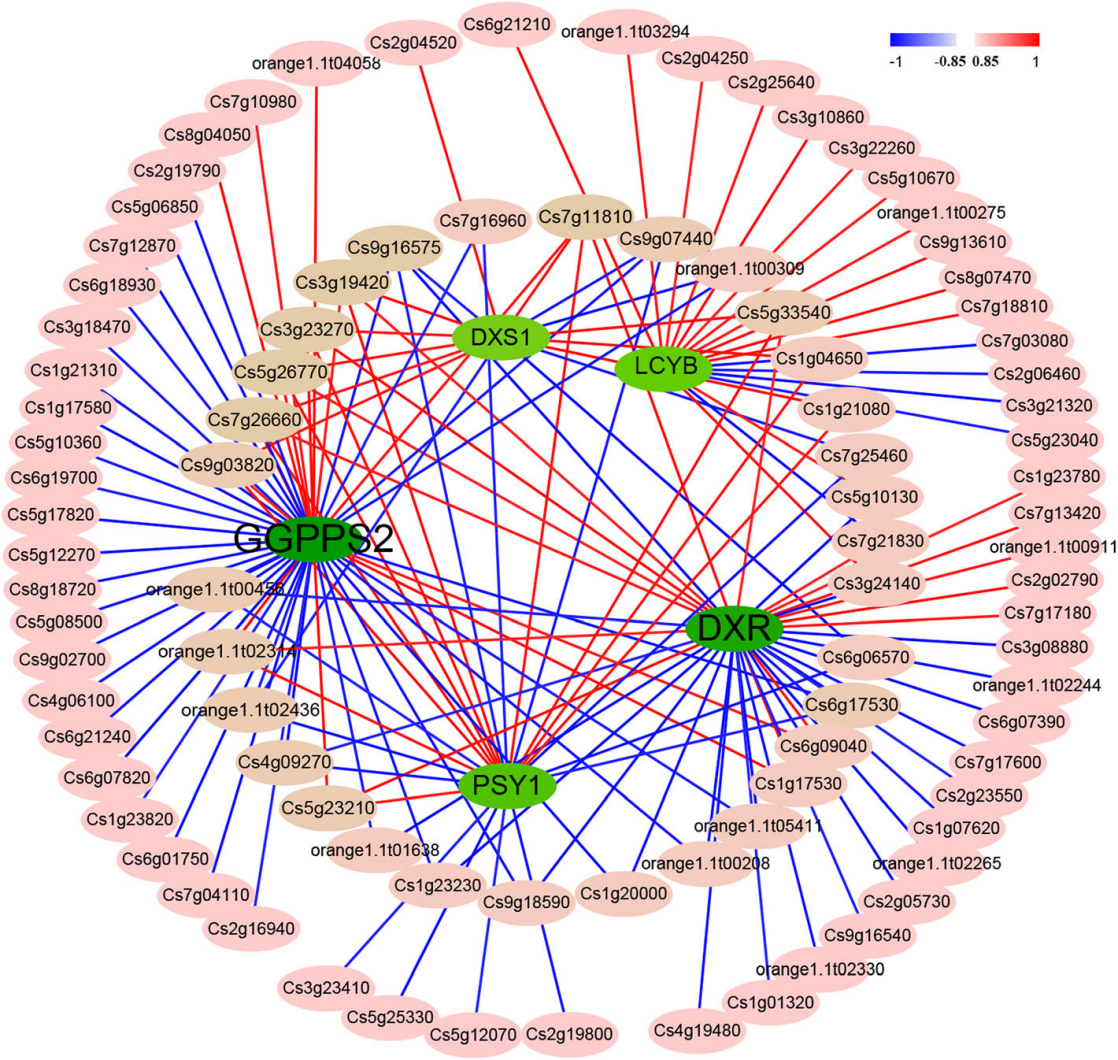
The relationships between *DXS1* (Cs1g20530), *DXR* (Cs5g05440), *GGPPS2* (Cs8g02140), *PSY1* (Cs6g15910) and *LCYB* (orange1.1 t00772) and its potential TFs based on transcriptome data

To better understand the potential role of TFs in the regulation of *DXS1*, *DXR*, *GGPPS2*, *LCYB* and *PSY1*, and thus contributing to lycopene accumulation, their promoters were used to predict the possible binding sites using PlantRegMap (Plant TF Database). Potentially 83, 147, 183, 104 and 228 TF binding site in *DXS1*, *DXR*, *GGPPS2*, *LCYB* and *PSY1* promoters were predicted, respectively (Additional file 2: Table S6). However, 16, 40, 48, 18 and 24 TFs had high Pearson correlation coefficients (|r|> 0.85), with 63 and 83 as potential positive and negative regulators respectively (Fig. 5; Additional file 2: Table S7). Among the 146 potential regulators, there were 93 TFs (17 TFs from AP2 family, 12 from MADS, 11 from GRAS, eight from MYB, five from TCP, WRKY and bZIP, four from SBP, three from ERF, HD-ZIP and NAC) (Additional file 2: Table S7).

Some TFs were highly positively or negatively correlated with *DXS1*, *DXR*, *GGPPS2*, *LCYB* and *PSY1* genes that contribute to carotenoids accumulation. These TFs might regulate the expression levels of five important genes that contribute to lycopene accumulation in CNO fruit. For example, *DXS1* was positively correlated with 10 TFs, and negatively correlated with six TFs, and the coefficients of seven TFs were higher than 0.900, while four were lower than − 0.900 (Fig. 5; Additional file 2: Table S7).

Some potential TFs bind to multiple genes, with high Pearson correlation coefficients. For example, Cs3g19420, Cs3g23270, Cs5g26720, Cs7g11810 and Cs7g26660 might positively regulate the four important pathway genes, while Cs9g16575 might be a negative regulator. Some TFs could regulate three of five pathway genes, such as Cs5g23210, Cs5g33540, Cs9g03820 and orange1.1 t02314 which might be potential positive regulators, while Cs4g09270, Cs6g17530, orange1.1 t00456, orange1.1 t02436 and Cs9g07440 might be negative ones (Fig. 5; Additional file 2: Table S7).

### Important pathway genes and TFs for flavonoids biosynthesis

The contents of flavonoids tended to decrease during fruit development (Fig. 1 B). The expression levels of the most flavonoids biosynthetic genes were found to be high at 60 DAF in CNO, while dropped at 120 and 150 DAF, including *PAL-1*, *PAL-3*, *4CL-2*, *4CL-5*, *CHS-1* and *CHI* (Additional file 4: Fig. S2). Results of Mfuzz analysis of Cluster1 showed that their expression tended to decrease (Fig. 3), and KEGG annotation found that the genes in the cluster were mainly involved in metabolic pathways, biosynthesis of secondary metabolites and phenylpropanoid biosynthesis (Additional file 5: Fig. S3). Coincidently, *PAL-1*, *PAL-3*, *4CL-2*, *4CL-5*, *CHS-1* and *CHI* grouped into Cluster1 were identified via gene annotation.

**Fig. 6:**
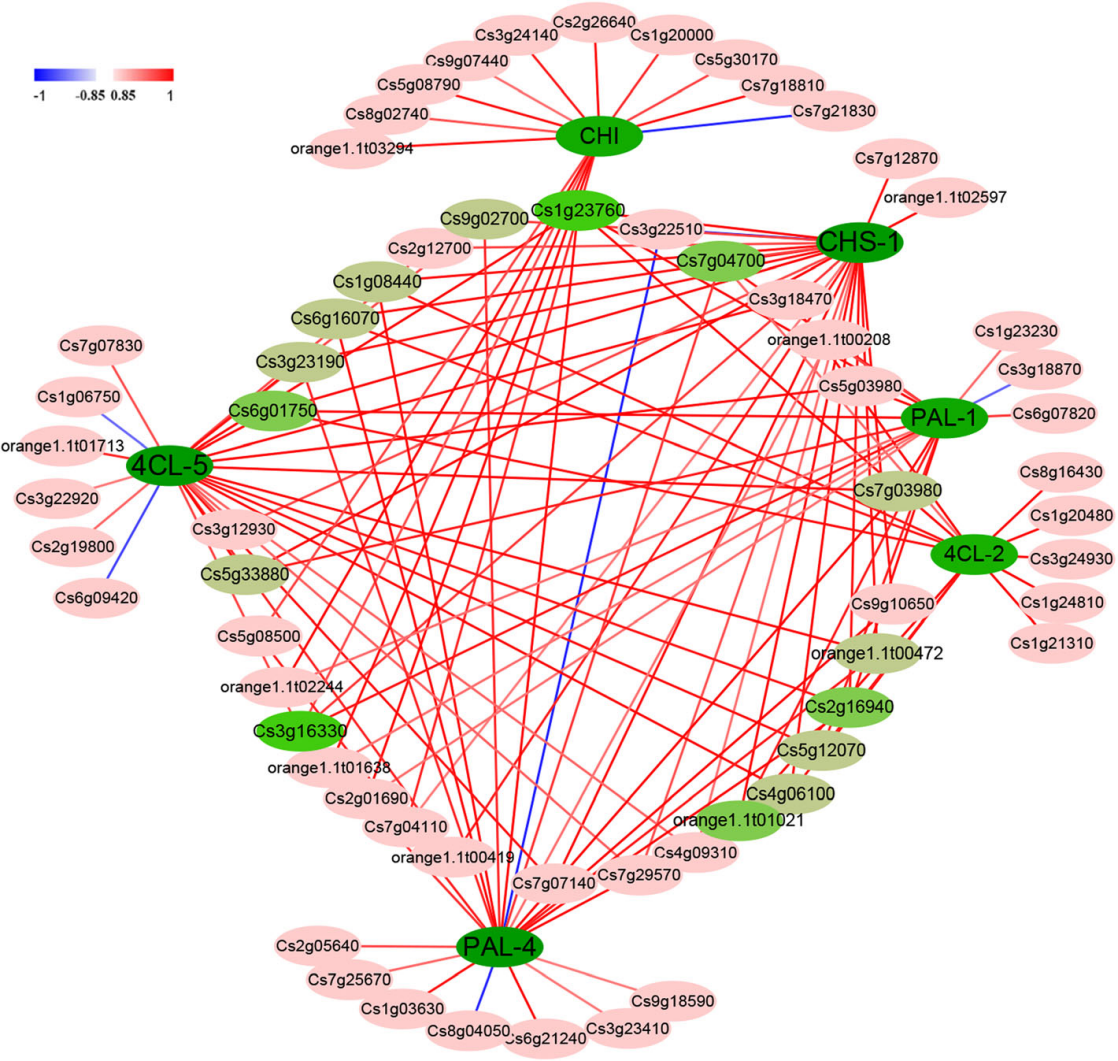
The relationships between *PAL-1* (Cs6g11940), *PAL-4* (Cs8g16290), *4CL-2* (Cs5g24900), *4CL-5* (orange1.1 t04489), *CHS-1* (Cs2g14720) and *CHI* (Cs7g28130) genes and its potential TFs based on transcriptome data

To further identify the potential TFs that may regulate the above six pathway genes and the accumulation of flavonoids, TFs were predicted using PlantRegMap. The results showed that there were potentially 213, 229, 95, 262, 227 and 102 TF binding sites in *PAL-1*, *PAL-3*, *4CL-2*, *4CL-5*, *CHS-1* and *CHI* promoters, respectively (Additional file 2: Table S8). The pathway genes with its potential 17, 22, 14, 25, 24 and 16 TFs were found to have high Pearson correlation coefficients (|r|> 0.85), with 111 and seven as potential positive and negative regulators, respectively (Fig. 6; Additional file 2: Table S9). Among the 118 potential regulators, there were 64 TFs, mainly including nine TFs from WRKY family, eight from MYB, six from bHLH and four from ERF (Additional file 2: Table S9).

Some TFs were highly positively or negatively correlated with *PAL-1*, *PAL-3*, *4CL-2*, *4CL-5*, *CHS-1* and *CHI* genes that contribute to flavonoids accumulation. These TFs might regulate the expression levels of the six important pathway genes that contribute to flavonoids accumulation in CNO fruit. For instance, PAL-1 was highly and positively correlated with 16 TFs, while negatively correlated with one TF, among them, the Pearson correlation coefficients of 12 TFs were higher than 0.900 (Fig. 6; Additional file 2: Table S9).

Among the potential TFs that had high Pearson correlation coefficients with the key pathway genes, Cs1g23760 and Cs3g16330 might positively regulate the five of six key pathway genes, while Cs2g16940, Cs7g04700, Cs6g01750 and orange1.1 t01021 might positively regulate the four out of six pathway genes, and nine TFs (Cs1g08440, Cs3g23190, Cs4g06100, Cs5g12070, Cs5g33880, Cs6g16070, Cs7g03980, Cs9g02700 and orange1.1 t00472) could bind three out of six pathway genes as positive regulators (Fig. 6; Additional file 2: Table S9). These TFs might play important roles in flavonoids biosynthesis.

### Postulated hierarchical network in regulating carotenoids and flavonoids profiles in CNO

Both sets of 15 candidate TFs could bind at least three carotenoids and flavonoids biosynthesis genes, respectively. Further analysis identified 24 TFs that could regulate important genes involved in both carotenoids and flavonoids biosynthesis pathways. Twenty of these TFs might positively and negatively regulate flavonoid biosynthesis and carotenogenesis genes, respectively, whereas two of them had the opposite effects and the other two were positively correlated with the important genes in both biosynthetic pathways (Additional file 6: Fig. S4). Thus, these TFs might directly regulate the genes related to the biosynthesis pathways for both carotenoids and flavonoids.

## Discussion

### The key stage for lycopene and carotenoids accumulation

Carotenoids were the important primary and secondary metabolites in plants [38]. Seven main carotenoids were detected in CNO juice sacs using HPLC (Additional file 1: Table S1), maybe because the intermediate lycopene was over-accumulated, which may affect the biosynthesis of downstream carotenoids and other branch products. Similar to previous result, lycopene was found to be the most abundant carotenoid in CNO [6]. In the study, 90–120 DAF was identified as the key stage for lycopene and carotenoid biosynthesis (Fig. 1a). However, 180 DAF was found to be the key stage for lycopene accumulation in a sweet orange, ‘Hong Anliu’ [20]. Thus, this study has for the first time revealed an early regulatory mechanism governing lycopene over-accumulation in CNO fruit.

### Important pathway genes contributing to carotenoids accumulation

In MEP pathway, GGPP is the direct precursor for the production of the first carotenoid, the colorless phytoene, catalyzed by PSY [8, 18]. However, *DXS* and *DXR* within MEP pathway play important roles in regulating the biosynthesis of carotenoid, abscisic acid, GAs and monoterpene [39]. To date, most of the carotenoid biosynthetic genes were cloned from citrus and other plants, such as *GGPPS*, *PSY*, *PDS*, *ZDS*, *CRTISO*, *LCYB*, *LCYE*, *DHY*, *ZED* and *CCD* [8, 12], among them, *PSY*, *PDS*, *ZDS* and *CRTISO* were upstream to lycopene biosynthesis, while *LCYB*, *LCYE*, *DHY*, *ZED* and *CCD* were downstream. It was reported that the expression level of *PSY* was related to carotenoids accumulation in tomato and citrus, and *PSY* played an important role in lycopene biosynthesis [17, 18]. *LCYB* and *LCYE* contributed to the production of β-carotene and α-carotene, respectively, using lycopene as a precursor [40]. The expression level of *PSY* in CNO was significantly higher than that in Washington navel orange fruit [41], and the higher expression levels of *DXR* and *GGPPS* were found in CNO fruit than in Washington navel orange fruit at the mature stage [29]. However, *PSY* and *LCYB* were found to be important for the accumulation of carotenoids in the ‘Hong Anliu’ sweet orange [17]. Previous studies showed that overexpression of *ZDS* led to a more accumulation of upstream carotenoids in tomato [42], and in our study, *ZDS3* expressed at 90 and 120 DAF stage, the key stage for lycopene and carotenoid biosynthesis, so we speculated that the *ZDS3* might contribute to lycopene accumulation in CNO.

In this study, the four genes, *DXS1*, *DXR*, *GGPPS2* and *PSY1* were found to significantly change during fruit development, based on metabolomic data, KEGG and Mfuzz cluster, and also showed a high Pearson correlation values (> 0.80) (Additional file 3: Fig. S1; Fig.2; Table 1), indicating the important contribution of these genes to carotenoids accumulation in CNO fruit. The expression levels of these four genes were the highest at 120 DAF, coinciding with the highest increased rate of lycopene level from 90 to 120 DAF (Fig. 3). Although another significant increase of lycopene content happened from 120 to 150 DAF with a slightly lower rate compared to the previous period, the expression levels of the four genes at 150 DAF were lower than those at 90 and 120 DAF.

It is known that the accumulation of carotenoids is a net effect of several biological processes, including synthesis, transportation and degradation. The *LCYE* and *LCYB* are key genes for the lycopene degradation [17, 43]. In our study, the *LCYB* expression maintained relative low level throughout fruit development (Fig. 3). Moreover, another lycopene accumulation-related gene *LCYE* was found to have a high Pearson correlation coefficient with lycopene content (Table 1). However, the contents of its direct products, α-carotene and lutein, were retained at low levels. Therefor, in the study, our data indicated that the relative low enzymatic activity of *LCYE* together with the lower *LCYB* expression level before 150 DAF was one of the most important reasons for the higher accumulation of lycopene.

Taken together, we speculated that high expression levels of *DXS1*, *DXR*, *GGPPS2*, *PSY1* and relative low expression level of *LCYB* might play important role for lycopene accumulation in CNO fruit.

### Potential TFs may play important roles in lycopene accumulation

TFs play important roles in plant biological regulatory networks. It was reported that some TFs regulated the biosynthesis of carotenoids. For example, CsMADS6 could bind *PSY* and *LCYB* promoters and directly regulate their expression levels, and thus affect the accumulation of carotenoids in sweet orange fruit, ‘Hong Anliu’ [17], while PIFs, HY5, RAP2.2, RIN and SlMADS1 can directly bind the PSY promoter to alter the carotenoids profiles [25, 26, 44]. In this study, 16, 40, 48, 24 and 18 TFs showed high Pearson correlation coefficients (|r|≥0.85) with *DXS1*, *DXR*, *GGPPS2*, *PSY1* and *LCYB* (Additional file 2: Table S7). The TFs were involved in AP2, GRAS, MADS, MYB and WRKY type transcription factors, were found to possibly bind *DXS1*, *DXR*, *GGPPS2* and *PSY1* promoters (Additional file 2: Table S7). Eight and seven TFs may positively and negatively regulate at least three carotenoids biosynthetic pathway genes respectively, and thus may play important roles in the over-accumulation of lycopene in CNO. It was reported that over-expression of *CubHLH1* decreased lycopene content in tomato, while *PpPLENA* had the opposite regulation effect [22, 24]. And some TFs could affect plant photosynthesis and result in carotenoid accumulation rather than directly bind carotenoid biosynthetic genes, such as Gloden2-like in tomato [24]. Therefore, the TFs identified in the study might regulate other biological processes, such as hormone signal transduction and photosynthesis to affect the special carotenoids profile in the CNO fruit. However, as reported in ‘Hong Anliu’, the Pearson correlation coefficient was only − 0.762 between CsMADS6 (Cs7g16970) and *PSY1*, while other MADS TFs, Cs7g11810 and Cs1g21080 showed higher and positive Pearson correlation coefficients (> 0.900) in CNO fruit, but it still need further confirmation by yeast one-hybrid (Y1H) and electrophoretic mobility shift assay (EMSA) study [17]. It would be of interest to find out if they are more important than CsMADS6 in lycopene accumulation.

### Pathway genes and TFs that may play important roles in flavonoids accumulation

Flavonoids are important secondary metabolites in citrus fruit, and biosynthesized via phenylpropanoid biosynthetic pathway, with *PAL*, *C4H*, *4CL*, *CHS* and *CHI* genes involved in the pathway [32]. The contents of various flavonoids were stable or decreased during fruit development, and the expression levels of *PAL*, *C4H*, *4CL*, *CHS-1* and *CHI* were especially-expressed at 60 DAF and significantly decreased at 90, 120 and 210 DAF in ‘Fenghuang’ pomelo, ‘KaoPan’ pomelo, ‘Huanong red’ pomelo and ‘HB’ pomelo [32]. Similarly in this study, the expression levels of *PAL-1*, *PAL-3*, *4CL-2*, *4CL-5*, *CHS-1* and *CHI* genes were found to decrease during fruit development (Additional file 4: Fig. S2), indicating the important roles of these genes for flavonoids accumulation at early stages.

Many TFs were reported to regulate phenylpropanoid biosynthetic pathway genes, and then affect the contents of anthocyanin, lignin and flavonoids. For example, MYBF1 was found to bind *CHS* promoter and then regulate the synthesis of flavonol in citrus and grapevine [34, 37], while many other MYB TFs were reported to bind *PAL*, *4CL* and *CHS* promoters and then regulate anthocyanin and lignin biosynthesis [44]. In the study, 64 potential TFs with high Pearson correlation coefficients were predicted based on *PAL-1*, *PAL-3*, *4CL-2*, *4CL-5*, *CHS-1* and *CHI* promoters’ analysis (Fig. 6). Some of them could potentially bind promoters of multiple genes, and thus may be most important TFs regulating flavonoids accumulation in CNO fruit.

### Potential TFs that may co-regulate carotenoids and flavonoids accumulation

In the study, in CNO fruit, the content of carotenoids and expression levels of key genes involved in carotenoids synthesis tended to increase, while the level of flavonoids and expression levels of the key genes tended to decrease (Fig. 1; Fig. 3). Further analysis revealed that both pathways’ key genes might be co-regulated by 24 potential TFs directly (Additional file 6: Fig. S4). For instance, Cs1g21310 might positively and negatively regulate flavonoids and carotenoids accumulation. Considering the potential TFs with direct functions in carotenoids and flavonoids biosynthesis, 24 TFs might serve as important TFs for the accumulation of carotenoids and flavonoids in CNO fruits to form a regulatory network (Additional file 6: Fig. S4). These TFs may form an integrative fruit quality regulatory network with various metabolic pathways in fruits.

## Conclusions

Carotenoids and flavonoids are important metabolites in citrus fruit, and lycopene was found to be the major compound contributing to the red flesh in CNO, while narirutin as the dominant flavonoid at the early stage. Carotenoids and flavonoids profiling of CNO juice sacs at six developmental stages identified 90–120 DAF as the key stage for the over-accumulation of lycopene. Transcriptomic analysis indicated that *DXS1*, *DXR*, *GGPPS2*, *PSY1* and *LCYB* genes were the most important genes for carotenoids accumulation, while *PAL-1*, *PAL-4*, *4CL-2*, *4CL-5*, *CHS-1* and *CHI* for flavonoids biosynthesis. Both sets of 15 candidate TFs were found to potentially bind the promoters of at least three carotenoids/flavonoids key pathway genes to regulate their expression levels, and thus help generate the special carotenoids and flavonoids profiles in CNO fruits. In addition, a total of 24 TFs were postulated as co-regulators in both pathways directly.

## Methods

### Materials

The fruit of CNO (*Citrus sinensis* Osbeck) were collected from the National Citrus Breeding Center (Wuhan, Hubei Province, China), the CNO cultivar was identified by the germplasm nursery manager of Zongzhou Xie. The fruit were harvested from the adult and healthy trees under normal management at six fruit development stages of 60, 90, 120, 150, 180 and 210 DAF. Three biological replicates per stage were analyzed, with each replicate consisting of six healthy and similar sizes fruit from three different plants. The juice sacs were immediately separated with a scalpel, and then placed in liquid nitrogen and stored at − 80 °C until analysis.

### Extraction and identification of carotenoids

Carotenoids were extracted from 2 g juice sacs and then measured according to Liu et al. [20]. Briefly, 2 g juice sacs were grounded into power in liquid nitrogen for the extraction. Carotenoids profiling were conducted by high-performance liquid chromatography (HPLC, Waters 1525 reverse-phase HPLC with a 2996 photodiode array detector) with a C30 carotenoid column (250 mm × 4.6 mm, YMC, Wilmington, NC, USA). The carotenoids were identified basing on the retention times and the absorption spectra with authentic standards according to our previous studies [20].

### Determination of flavonoids

Flavonoids were extracted from 1 g juice sacs for each sample using the method according to Chen et al. [9] with minor modification. The samples were grounded into power in liquid nitrogen, 1 g power were then used to flavonpids extraction in 2 mL 80% aqueous methanol for 60 min at 40 °C in ultrasonic bath model FS60 (Fisher Scientific, Pittsburgh, PA). The extraction were centrifuged at 10,000×g for 10 min, then the supernatant was filtered through a 0.22 μm micropore and analyzed using HPLC. Flavonoids were analyzed using the HPLC system with a C18 Hypersil GOLD column (250 mm × 4.6 mm × 5 μm, Thermo Scientific). The parameters for HPLC of flavonoids were according Chen et al. [9].

### RNA extraction, Transcriptomic sequencing and analysis

The CNO juice sacs at 60, 90, 120 and 150 DAF stages were used to total RNA extraction as described by Liu et al. [45], 2 μg total RNA for each sample was used for cDNA library construction according to the protocol (TruSeq RNA Sample Preparation v2 Guide, Illumina, United States). The mRNA was purified using the Illumina TruSeq RNA Sample Prep Kit v2, and then assessed using the Agilent Technologies 2100 Bioanalyzer (Agilent, United States). The first- and second-strand complementary DNA was synthesized, double-strand cDNA was purified, the adapters were added, and then the mRNA was cleaved into short fragments of approximately 270 bp. The constructed libraries were sequenced on an Illumina HiSeq 4000 platform in a paired-end (2 × 100 bp) mode by Millennium Co. (Seoul, Korea). The RNA-seq raw-data were uploaded onto NCBI (SRA) under the accession number PRJNA636131.

The genome and annotation files of Valencia orange (*C. sinensis*) were downloaded from the Orange Genome Annotation Project (http://citrus.hzau.edu.cn/orange/download/ index.php). A reference index by STAR (version 2.7) was built. Raw data were filtered using Trimmomatic (0.36) under default parameters, then the clean data were mapped to reference genome using STAR, and the counts calculated using Htseq-count based on mapping results. The edgeR was used to determine the differentially expressed genes, and then the DEGs were used for function annotation based on the Mercator sequence annotation software (https://www.plabipd.de/portal/web/guest/home1). The DEGs were subjected to KEGG enrichment analysis and GO annotation using KOBAS 3.0 (http://kobas.cbi.pku.edu.cn/) and TBtools [46], respectively. The expressed genes were clustered into nine groups using the Mfuzz package in R.

### Prediction of potential binding TF

The promoter sequences of *DXS1* (Cs1g20530), *DXR* (Cs5g05440), *GGPPS2* (Cs8g02140), *PSY1* (Cs6g15910), *LCYB* (orange1.1 t00772), *PAL-1* (Cs6g11940), *PAL-4* (Cs8g16290), *4CL-2* (Cs5g24900), *4CL-5* (orange1.1 t04489), *CHS-1* (Cs2g14720) and *CHI* (Cs7g28130) were used to predict the binding TFs using the Regulation Prediction function in PlantRegMap (http://plantregmap.cbi.pku.edu.cn/index.php). The *C. sinensis* database was used as a library, and *P*-value was lower than 1e^− 5^. The TFs were converted to the similar genes in Valencia sweet orange genome using BLAST function. All of the TFs in MADS, AP2 and GRAS (based on Mercator v.3.6 annotation results) were selected for further analysis. Pearson correlation coefficients between the above pathway genes with potential binding TFs were calculated, based on transcriptome data by R or function with ‘Pearson’ method. The Pearson correlation coefficient value was filtered by higher than 0.850 or lower than − 0.850, and the results were shown using Cytoscapev3.7.2.

### Data analysis

The contents of carotenoids and flavonoids were calculated using the standard curves. The R packages (nortest, stats and pgirmess) were used for analysis of variance with ANOVA (*P*< 0.05). Bar chart and dot charts were made based on DEGs and KEGG analysis results using R package, ggplot2. A heatmap of carotenoids biosynthesis-related genes was constructed based on transcriptome data using TBtools [46].

## Supplementary Information

**Additional file 1: Table S1.** Carotenoids identified in CNO at six developmental stages (μg/g, FW). U: undetectable. **Table S2.** Flavonoids identified in CNO at six developmental stages (ng/g, FW). U: undetectable. **Table S3.** Basic statistics of RNA-Seq clean-data mapped to the reference genome. **Table S4.** The expression levels of genes in CNO fruit (FPKM). **Table S5.** The expressed genes were clustered into nine groups by Mfuzz.

**Additional file 2: Table S6.** TFs were predicted in DXS1, DXR, GGPPS2, PSY1 and LCYB. **Table S7.** The TFs that showed high Pearson correlation coefficients with carotenoids synthesis-related genes. PCC^a^: Pearson correlation coefficient. **Table S8.** TFs predicted in *PAL-1, PAL-4, 4CL-2, 4CL-5, CHS-1* and *CHI* genes. **Table S9.** The TFs that showed high Pearson correlation coefficients with flavonoids synthesis-related genes. PCC^a^: Pearson correlation coefficient. **Table S10.** TFs predicted in 30 most important TFs that regulate at least three carotenoid or flavonoids genes.

**Additional file 3: Figure S1.** Differentially expressed genes in juice sacs of CNO fruit at different development stages. A, Numbers of differentially expressed genes at different developmental stages. B, KEGG analysis result of the differentially expressed genes in juice sacs between 90 DAF and 120 DAF.

**Additional file 4: Figure S2.** A heatmap showing the expression levels of flavonoids biosynthesis-related genes in CNO fruit based on transcriptome data.

**Additional file 5: Figure S3.** KEGG analysis result of the differentially expressed genes in juice sacs between 60 DAF and 90 DAF.

**Additional file 6: Figure S4.** TFs that regulated the carotenoids and flavonoids biosynthesis genes.

## Data Availability

All data generated or analyzed during this study were included in this published article and the additional files. The RNA-seq data are available from the NCBI under the accession number PRJNA636131.

## Abbreviations

DXS: 1-deoxy-D-xylulose-5-phosphate synthase
DXR: Deoxyxylulose 5-phosphate reductoisomerase
GGPPS: Geranylgeranyl diphosphate synthase
PSY: Phytoene synthase
LCYB: Lycopene β-cyclase
LCYE: Lycopene epsilon-cyclase
PAL: Phenylalanine ammonia-lyase
4CL: 4-Coumarate-CoA ligase
CHS: Chalcone synthase
CHI: Chalcone isomerase
CNO: Cara cara navel orange
TFs: Transcription factors
DAF: Days after florescence
