# Supplementary figures and images for: Integrated Transcriptomic and Metabolomic analysis reveals a transcriptional regulation network for the biosynthesis of carotenoids and flavonoids in ‘Cara cara’ navel Orange
Source: BMC Plant Biol. 2021 Jan 7;21:29. doi: 10.1186/s12870-020-02808-3 (PMC7792078; doi:10.1186/s12870-020-02808-3)

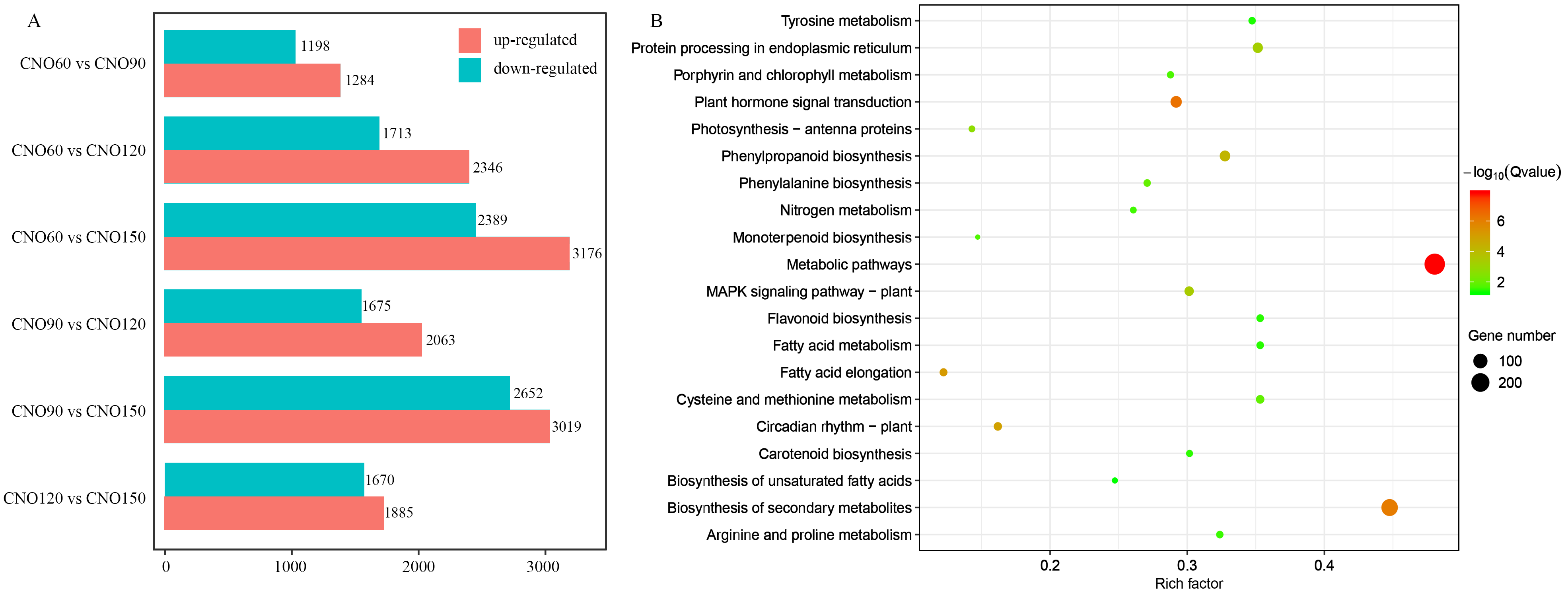

Supplement: Supplementary file 3 — Additional file 3: Figure S1. Differentially expressed genes in juice sacs of CNO fruit at different development stages. A, Numbers of differentially expressed genes at different developmental stages. B, KEGG analysis result of the differentially expressed genes in juice sacs between 90 DAF and 120 DAF. [file 12870_2020_2808_MOESM3_ESM.tif]

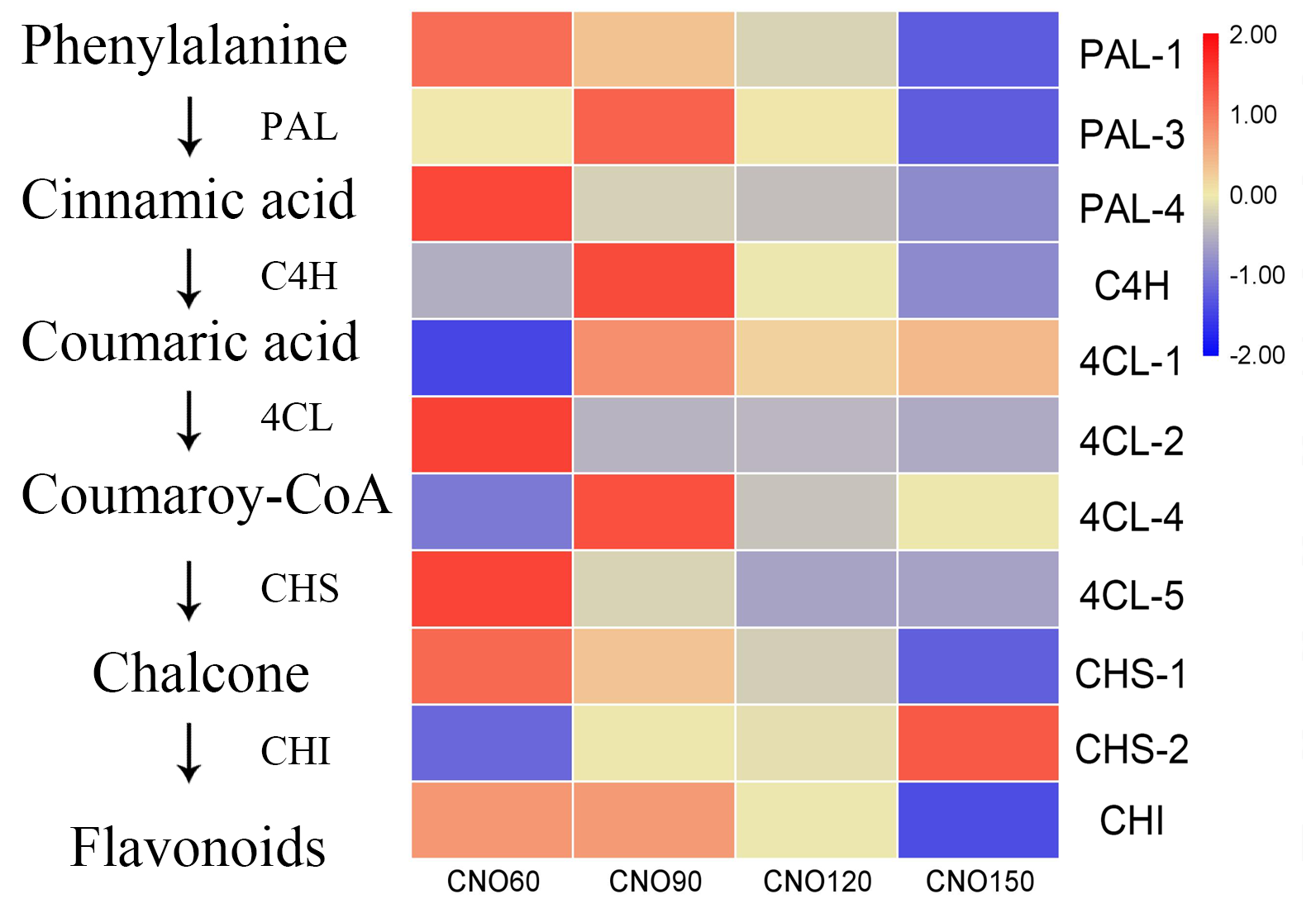

Supplement: Supplementary file 4 — Additional file 4: Figure S2. A heatmap showing the expression levels of flavonoids biosynthesis-related genes in CNO fruit based on transcriptome data. [file 12870_2020_2808_MOESM4_ESM.tif]

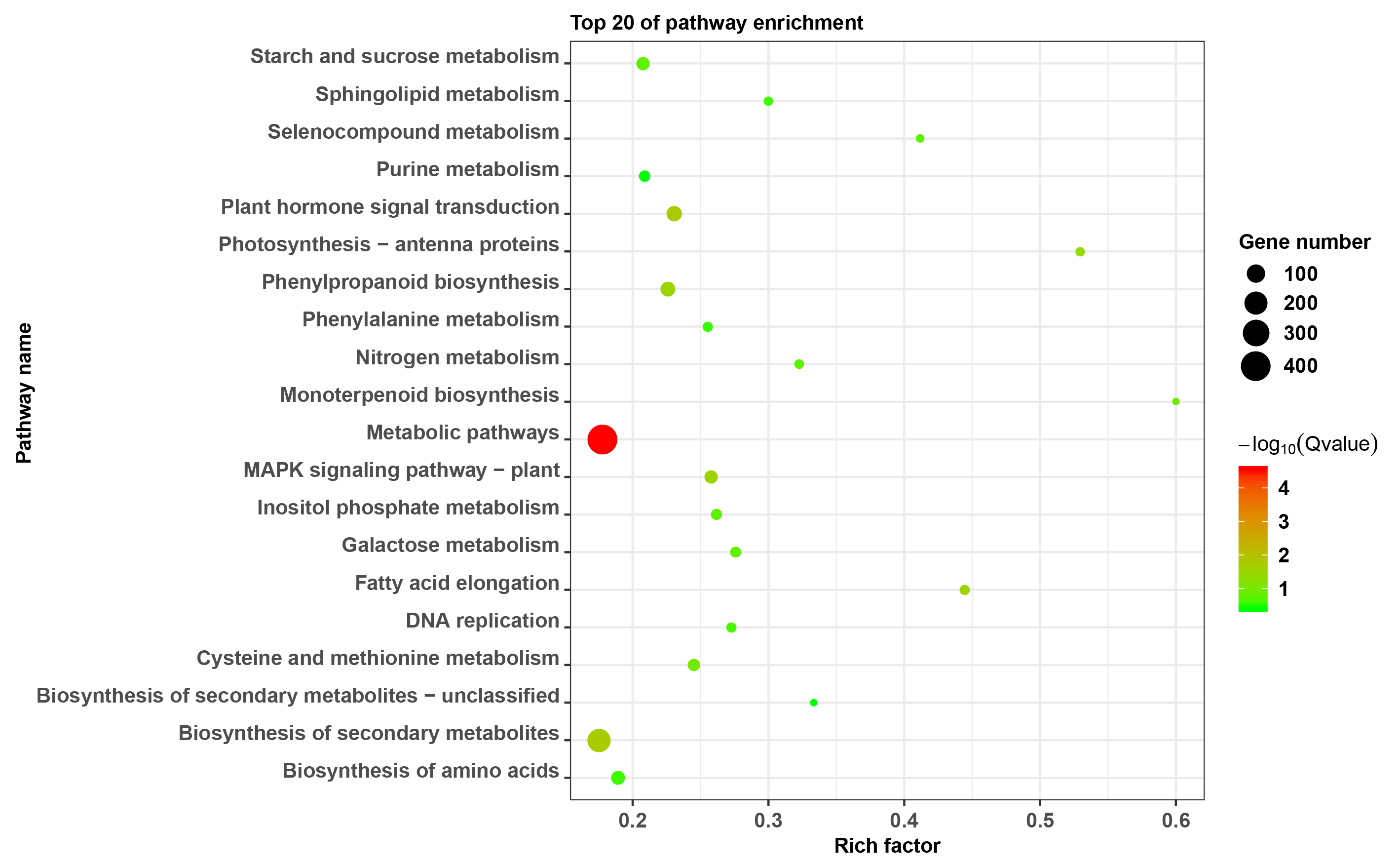

Supplement: Supplementary file 5 — Additional file 5: Figure S3. KEGG analysis result of the differentially expressed genes in juice sacs between 60 DAF and 90 DAF. [file 12870_2020_2808_MOESM5_ESM.tif]

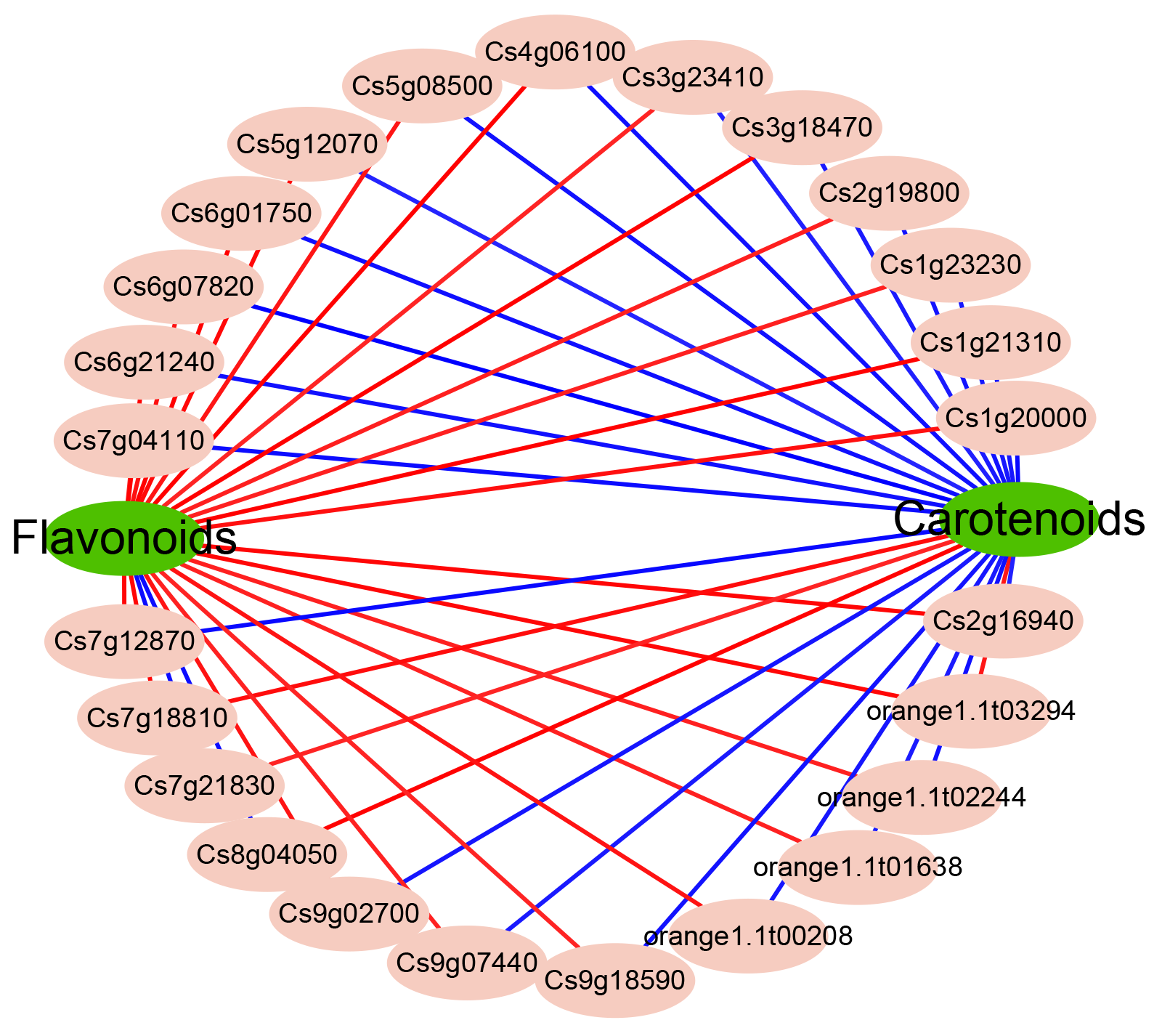

Supplement: Supplementary file 6 — Additional file 6: Figure S4. TFs that regulated the carotenoids and flavonoids biosynthesis genes. [file 12870_2020_2808_MOESM6_ESM.tif]
